# Domestic

**Published:** 1841-12-04

**Authors:** 


### DOMESTIC.

The author of the following letter, extracted from the Boston Medical and Surgical Journal, Dec. 1st,—John C. Martin, M. D.,—is by another represented as having undergone a most disgraceful and public prosecution, in consequence of his experiments, compelling him to fly his residence in Attleborough, Mass., fortunately to find a more profitable location in Greenville, Illinois. The letter was written in 1835, and the experiments contained in it were performed in consequence of hearing of those of Dr. Creely in England.

*Sir*,—The following experiments may not be uninteresting to you. They were undertaken for the public good and for the benefit of science. And although I have suffered severely in mind and in purse for making them, yet I am not sorry that the act has been committed; and all I regret is, that I am not located in a community, and surrounded by medical men, who can duly appreciate my motives, and encourage me in prosecuting a series of experiments which, I feel convinced, might lead to successful and happy results.

A case of small-pox, in its worst form, having appeared in Attleborough, where I reside, and having myself, like many other physicians, failed in obtaining fresh and pure vaccine virus, and having, moreover, witnessed and read of the frequent failures of the vaccine disease, as an antidote to the attacks of small-pox, I became exceedingly desirous of obtaining the virus directly from the cow. It is true that the source of the cow-pox virus is, and always has been, a matter of theory. Jenner, in his time, and many physicians of later times, imagined, and supposed themselves to have proved, that pure vaccine matter was the result of the action of small-pox in the cow. I have been anxious to determine this point, so that, should the theory prove to be true, physicians of this country might have it in their power at all times to obtain matter which would prove to be a more perfect prophylactic against variolous poison, than that which they are now obliged to use.

In order to test the theory fairly, I purchased a fat, healthy cow, eight years old, shut her in a stable, and fed her scantily for a few days. I then obtained some variolous matter from the individual who was sick with the small-pox, and who had been labouring under the disease eleven days. With this matter I inoculated the cow on the 2d day of October, 1835, in the following manner. I made, with a common lancet, fourteen or fifteen punctures in one of her teats, between the cuticle and true skin, taking care not to draw blood. I then inserted into these various punctures, quills charged with the variolous virus. The wounds soon disappeared, and presented no appearance of being variolated until five days. On the *fifth* day the animal seemed to show some indisposition, and on examining her teat I discovered one small elevated spot at the point of insertion of one of the quills, and an evident febrile heat in the teat, when compared with those not inoculated. This increased febrile heat continued for about forty-eight hours, and then subsided. During this time the animal lost her appetite, became thirsty, her milk ceased to be secreted, and her teat began to swell.

On the *eighth* and *ninth* days, a regular pustule was formed at the point inoculated, the margin of which contained some thin, light straw-coloured fluid. On the *tenth* day, the pustule had increased in size and become more prominent, and was distended with matter. At this period it was not regularly round, but presented an uneven surface. On the *eleventh* day, an evident change had taken place in the appearance of the pustule, it having begun suddenly to dry up. On the *thirteenth* day, the virus had become solid, so that the pustule was converted into a crust, or scab, of a dark-brown colour.

Besides introducing the small-pox virus into the udder, I inserted some also into a puncture which I made on the hip of the animal. This resulted in a sore, and in the falling off of the hair. This inoculation produced no pustule or eruption, save at the point of insertion, so far as I could discover.

I now determined to insert some of this new generated matter into the human system, and observe its effects. Accordingly, I selected a healthy boy, aged 10 years, for the subject of my first experiment; and on the evening of the 12th day of October, (the day I took some virus from the cow, being the tenth day of the existence of the pustule,) I inserted some of this virus into the boy's arm, in the same manner as in practising common vaccination. The symptoms resulting from the operation were the following: The virus lay dormant four days. On the *fifth* day, a slight inflammation or red spot arose around the point of insertion. From this period the vesicle ran its course, like the common vaccine vesicle, was characterized by a well-formed and regular areola, and in due time was transformed into a perfectly sound, mahogany-coloured scab. The boy exhibited but little indisposition during the course of the disease, except headach, and he continued to play with his fellows about the street, and I saw no symptoms in his case which do not attend the vaccine disease in its various stages. It should be mentioned, however, that two or three small pimples appeared on the boy's face and arm. These did not fill, but soon dried and disappeared.

While observing the rise and progress of this disease, I had no doubt that the eruption was like, and that it was, the true and perfect vaccine vesicle. In order that I might not be deceived, however, I took the boy to Providence, and exhibited his arm to two physicians of that place, Drs. Brownell and Toby, both of whom pronounced the eruption to be perfect vaccine, and gave me their opinion in writing to this effect. Having satisfied myself of the nature of the eruption produced in the boy's arm, I took matter from it on the *seventh* day, and inserted some of it into the arm of my own child, which was five months old. On the *fourth* day, a red spot appeared around the point of insertion, a vesicle was formed, and observed the same course and presented the same appearances as did that on the boy's arm, from which the virus was taken. The areola, perhaps, was not quite so regular as in the case of the boy; and the febrile excitement was greater in the child, which I attributed to its natural irritability of temperament. There were in this case a number of secondary eruptions on the surface, resulting from the vaccination.

The *third subject* vaccinated was also an infant. This child I vaccinated with matter from my child's arm, on the seventh day of the disease. This child's symptoms were similar to those presented by my own child; they were, perhaps, rather more severe. The areola was not so perfect, and there appeared on it a greater number of secondary vesicles, which became filled with fluid. The *fourth* individual vaccinated was a boy, four years old. The virus with which I vaccinated him was taken from the arm of the child whose case I last described. The symptoms attending this case were similar to those presented in the preceding one, except that they were more severe. The areola, however, was not so regular, and the vesicle was rather more imperfect, and a greater number of secondary eruptions presented themselves on the body, some of which filled and formed crusts.

I will not trouble you further in describing cases. The whole number of persons I vaccinated was twenty-three, and the cases above described will give you a notion of the character and progress of the others. I will remark, however, that I think the last individual vaccinated had the disease more severely, as the matter used in producing it was more remotely related to the cow.

Such have been the results of my experiments, and I should feel highly gratified and honoured with your opinion respecting them.

Very truly your friend,

J. C. Martin.

